# Fusariosis: An Invasive Fungal Disease in a Neutropenic Patient With Acute Myeloid Leukemia

**DOI:** 10.7759/cureus.29303

**Published:** 2022-09-18

**Authors:** Denis D Asiimwe, Malleswari Ravi, Carmen Isache

**Affiliations:** 1 Internal Medicine/Infectious Disease, University of Florida College of Medicine- Jacksonville, Jacksonville, USA; 2 Infectious Disease, University of Florida College of Medicine- Jacksonville, Jacksonville, USA

**Keywords:** mortality, immunosuppressed, acute myeloid leukemia (aml), profound neutropenia, fusarium

## Abstract

Although rare in the U.S invasive Fusariosis (IF) is increasingly being recognized as a cause of severe invasive fungal disease in patients with neutropenia in the setting of hematologic malignancy and hematopoietic stem cell transplants (HSCT). IF in these patients is associated with high mortality, moreover there are no guidelines on effective therapy, thus early diagnosis and involvement of an expert with experience in treating Fusariosis are imperative. We present a case of IF in a patient with profound prolonged neutropenia in the setting of chemotherapy for relapsed, refractory acute myeloid leukemia. A 33-year-old woman with relapsed acute myeloid leukemia (AML) was hospitalized for re-induction chemotherapy. Five days post cycle 1 she became neutropenic. She was treated with prophylactic antimicrobials that included acyclovir, levofloxacin, and Posaconazole. On day sixty she began to run a high-grade fever. The physical exam was remarkable for a temperature of 102 degrees Fahrenheit and a heart rate of 116 beats per minute. Complete blood count was remarkable for 130 WBC/ml, Hb 6.5 g/dl, hematocrit (HCT) 18.7%, 13000 platelets/ml, absolute neutrophils counts (ANC) of 0. Her CT chest showed new bilateral lung nodules. Antibiotics were changed to cefepime, vancomycin, and metronidazole on day sixty-two without response. On day sixty-five meropenem was started and cefepime stopped. On day sixty-eight posaconazole was stopped and amphotericin B was started and two days later fever became low grade. She developed hyperpigmented skin lesions with necrotic centers on extremities that were biopsied. Histopathology staining favored the presence of rare fungal hyphae. The culture of the biopsy sample grew *Fusarium spp* that was identified by DNA sequencing as *Fusarium falciforme*. Voriconazole and terbinafine were added. Her fevers resolved within the next 24 hours and she remained afebrile. *Fusarium* is a hyaline mold present in the environment. Infection is acquired by inoculation into the skin, intravascular devices, or inhalation. IF incidence is low in the United States. *F. solani* and *F. oxysporum* are the most predominant disease-causing species complexes. Invasive Fusariosis (IF) is a rare disease seen in patients with hematologic malignancy and hematopoietic stem cell transplants (HSCT) with profound neutropenia. Immunocompromised patients suffer disseminated disease to multiple sites as in this case, with mortality rates of between sixty to eighty percent in this patient population. Blood and skin lesions biopsy cultures are diagnostic. Blood cultures are positive in up to sixty percent of cases in about four days. Polymerase chain reaction (PCR) can identify *Fusarium* but species identification by PCR is difficult. Newer molecular methods are better for species identification. Histopathology can be helpful. Differential diagnoses include invasive aspergillosis (IA), mucormycosis, mycobacterial and dimorphic fungal infections. There are no guidelines for standard therapy. Amphotericin B or voriconazole are preferred. Combination therapy may be indicated. Neutrophil recovery is crucial. Adjunctive and preventive measures have roles.

## Introduction

Although rare in the U.S Invasive Fusariosis (IF) is increasingly being recognized as a cause of invasive fungal disease in patients with neutropenia in the setting of hematologic malignancy and hematocrit (HCT) [1,2]. IF in these patients is associated with high morbidity and mortality, moreover there are no guidelines on effective therapy, thus early diagnosis and consultation with an expert experienced in treating Fusariosis are imperative [1,2,3]. We present a case of a young woman with relapsed acute myeloid leukemia (AML) and prolonged neutropenia with fever due to IF.

## Case presentation

A 33-year-old woman with a history of acute myeloid leukemia (AML) was admitted to our hospital for re-induction chemotherapy following relapse six months after initial therapy. Five days following her first re-induction cycle of azacitidine and venetoclax she became neutropenic. She was prophylactically treated with acyclovir, levofloxacin , and posaconazole once she became neutropenic. On day sixty post cycle 1 she started running a high-grade fever. On physical exam, she was acutely ill appearing with alopecia, febrile to 102.0 degrees Fahrenheit and tachycardic with 116 beats per minute. She had mild tenderness over her left maxillary sinus. The rest of her initial physical exam was unremarkable. Laboratory work-up showed: white blood cells (WBC) 130 cells/ml, hemoglobin (Hb) 6.5 g/dl, hematocrit 18.7%, mean corpuscular volume (MCV) 85.8 Fl, platelets were 13,000 cells/ml, absolute neutrophil count (ANC) 0.0 cells/microliter. Sodium 135 mmol/L, potassium 3.4 mmol/L, chloride 104 mmol/L, bicarbonate 20 mmol/L, blood urea nitrogen 11 mg/dl, creatinine 1.0 mg/dl aspartate aminotransferase (AST) 15 IU/L, alanine transaminase (ALT) 20 IU/L, Alkaline phosphatase 151 IU/L, bilirubin 1.5 mg/dl, magnesium 1.6 mg/dl.

**Figure 1:**
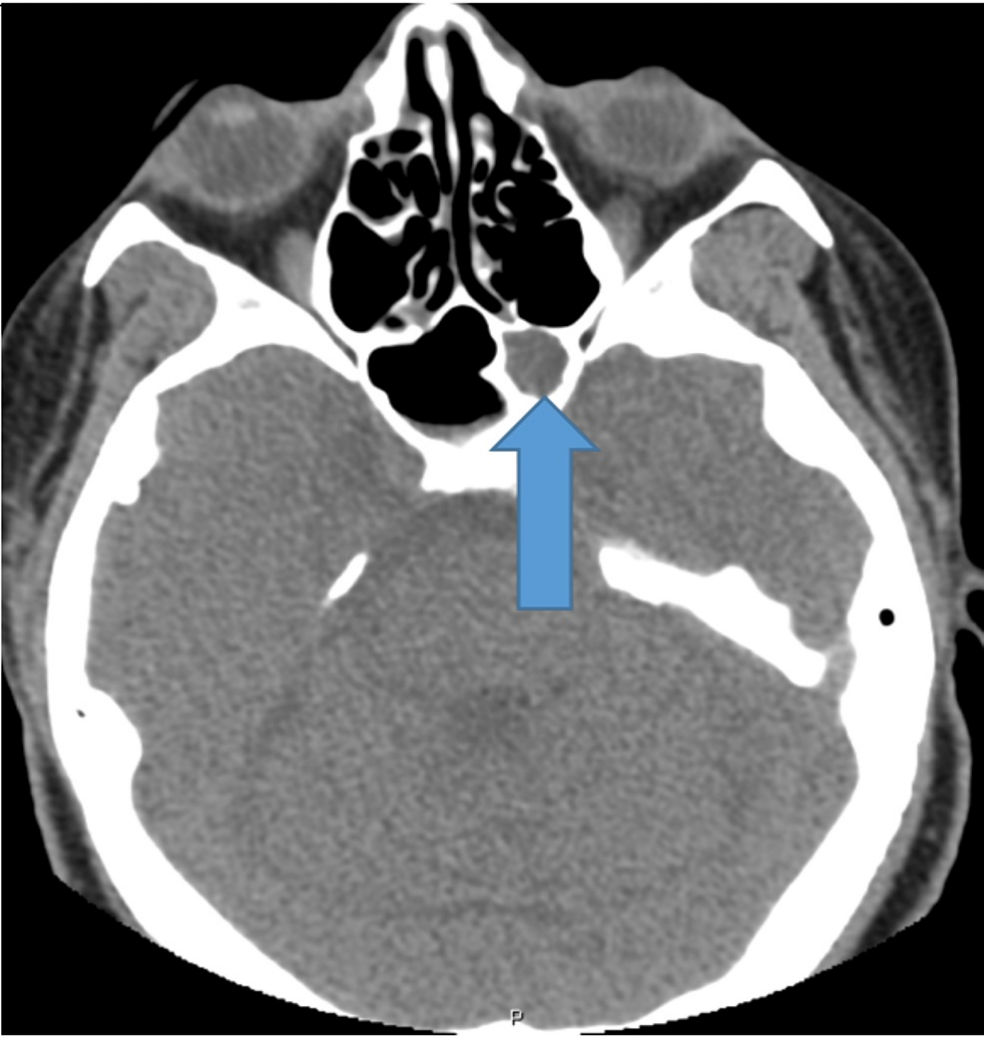
CT maxillofacial with blue arrow pointing to opacified left sphenoid sinus

**Figure 2:**
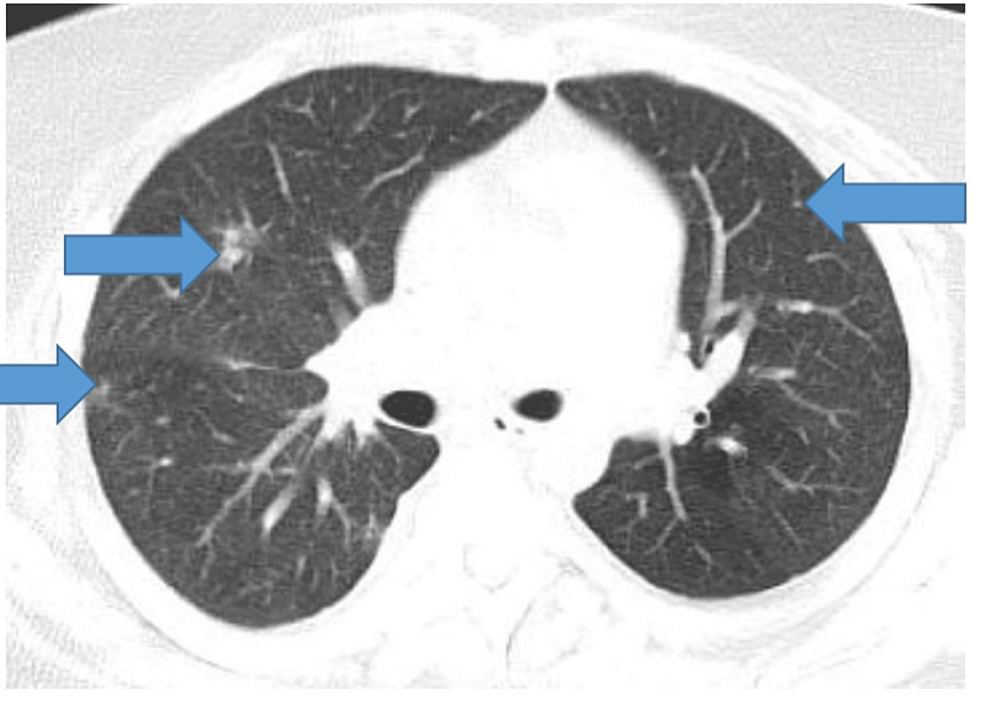
CT chest with arrows pointing to bilateral pulmonary nodules

Her maxillofacial computed tomography (CT) scan showed paranasal sinusitis of the left sphenoid sinus with occlusion of the left sphenoethmoidal recess (Figure 1). CT scan of her chest showed new multiple bilateral pulmonary nodules (Figure 2) and; the CT abdomen and pelvis were unrevealing. Routine and fungal blood cultures, Histoplasma urine and serum antigens, serum galactomannan, and QuantiFeron TB Gold test were all negative. 

**Figure 3:**
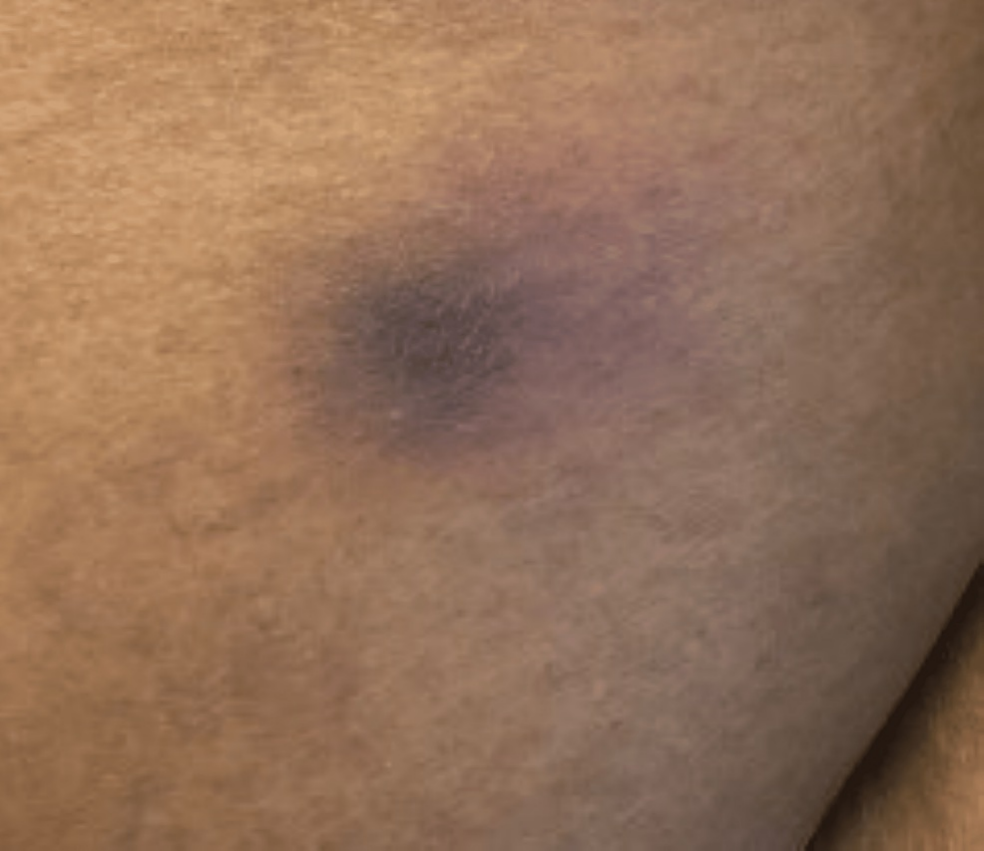
Initial skin lesions with hyperpigmented center

**Figure 4:**
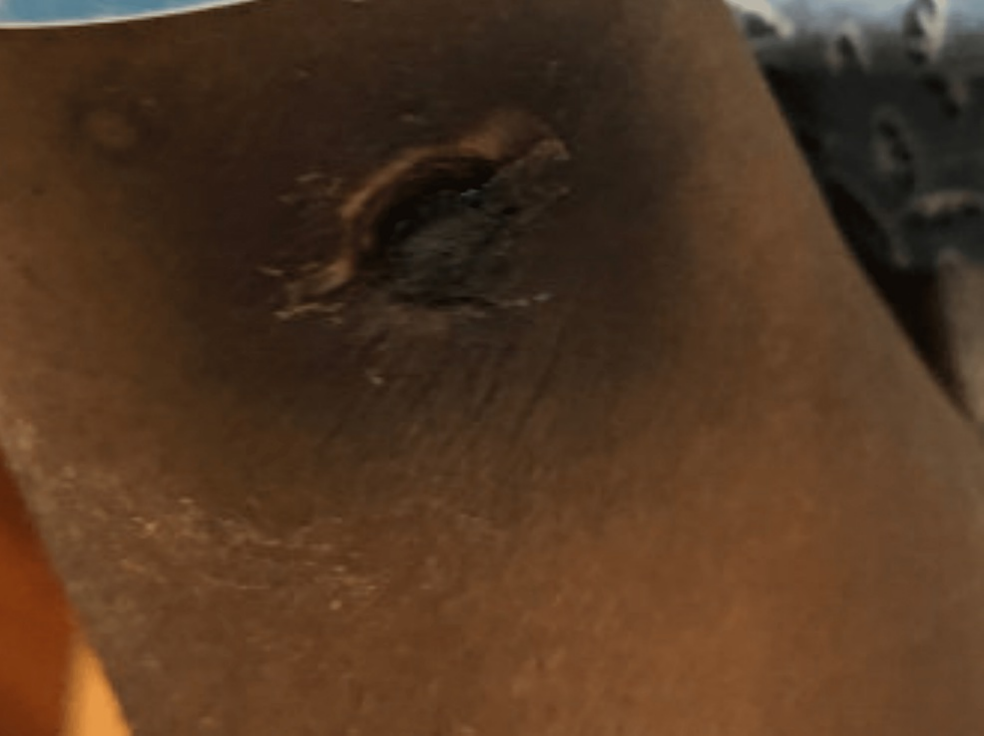
Skin lesions progressed to nodules with a necrotic center after about five days following initial development

Levofloxacon was discontinued and cefepime, vancomycin, and metronidazole were added. Paranasal sinus endoscopy was performed and showed mucosal crusting in the left sphenoid sinus without lesions or purulence; the specimen collected for cultures was negative however no specimen was sent for histopathology exam. Bronchoscopy was performed and her airways were normal. Bronchoalveolar lavage (BAL) specimen testing was negative for galactomannan, Pneumocystis jirovecii by Direct Fluorescent Antibody testing (DFA), fungus (smears and cultures), and acid-fast bacilli (smears, cultures, and MTB PCR). A biopsy of the pulmonary nodules was considered but deferred as the patient was at a very high risk of developing complications post-procedure. The patient continued to have fevers and at this point, cefepime was stopped and meropenem added, however, forty-eight hours later she continued to run a high-grade fever. At this point, her total serum bilirubin had increased to 3.5 mg/dl without an increase in transaminases. Ultrasound of the liver was performed and was unremarkable. Posaconazole was thought to be the cause of increased bilirubin and so it was stopped, and liposomal amphotericin B was added. Bilirubin normalized within one week after stopping posaconazole. Her profound neutropenia persisted. Forty-eight hours after starting amphotericin B fever became low grade however by this time the patient had developed multiple hyperpigmented skin lesions with necrotic centers on her extremities and abdomen. The lesions started as macules with hyperpigmented centers (Figure 3) and progressed to nodular lesions with necrotic centers (Figure 4). 

**Figure 5:**
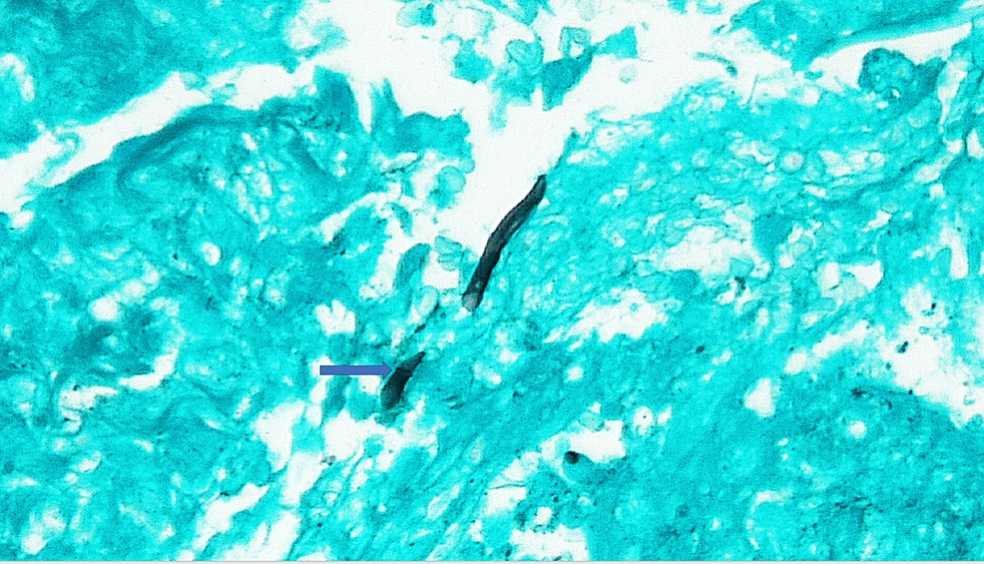
GMS stain of skin biopsy was reported as the equivocal but favored the presence of fungal hyphae shown by arrow

A skin biopsy was performed, and gomori methenamine silver (GMS) staining was reported as the equivocal but favored presence of rare fungal hyphae (Figure 5). Calcofluor staining showed septate hyphae. Culture of skin biopsy on inhibitory mold agar (IMA) and Mycosel agar grew mold in four days. The mold was identified using lactophenol cotton blue mount as *Fusarium* species. The specimen was sent to the University of Texas Health (UT Health), South Texas reference laboratory for further identification and antifungal susceptibility testing. Meanwhile, voriconazole and terbinafine were added to her antimicrobial regimen. Within 24 hours the fever resolved. 

**Figure 6:**
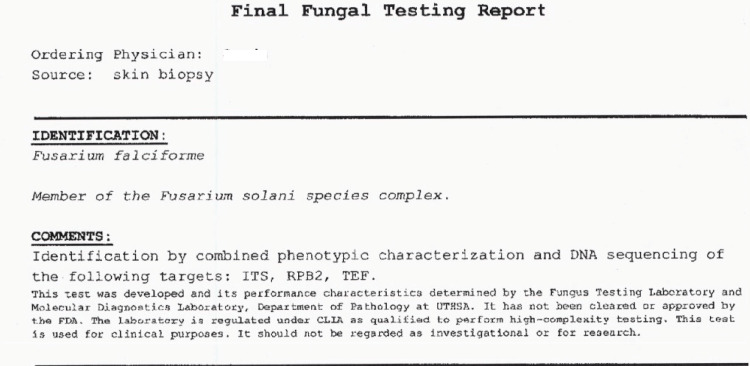
Final fungal identification report from UT Health, Southern Texas reference laboratory describing methods used to identify Fusarium species from biopsy of skin lesions obtained from our patient

**Figure 7:**
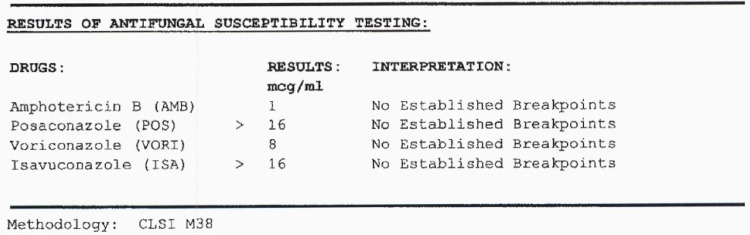
Results of antifungal susceptibility testing performed at UT Health, South Texas reference laboratory showing the MICs of amphotericn B, posaconazole, voriconazole and isavuconazole to Fusarium falciforme MIC - Minimum Inhibitory Concentration, CLSI- clinical Laboratory Standards Institute

At UT Health they used combined phenotypic characteristics and DNA sequencing methods to identify the mold as F.*falciforme*, a member of the F.*solani* species complex (see Figure 6 for the attached report). Antifungal susceptibility testing results are attached in Figure 7. 

The patient’s skin lesions completely resolved within fourteen days of starting treatment. Unfortunately, leukemia was refractory to all treatments and the patient's care was transitioned to comfort measures under hospice following goals of care discussions with the patient and her family. The patient remained afebrile through the rest of her hospitalization and was eventually discharged on day 98 on oral voriconazole and terbinafine. Oral agents were preferred to IV amphotericin B so as to avoid a central line, blood work monitoring, and patient comfort. No further imaging was obtained to assess pulmonary nodules due to the patient being on comfort measures. She died four months after discharge from the hospital. 

## Discussion

*Fusarium *is a hyaline mold that is widely distributed in the environment including in some plants, soil, and water [2]. *Fusarium *form biofilms in hospital water systems [2]. Human IF is a rare but emerging entity in patients with acute leukemia and HSCT with profound and prolonged neutropenia [2]. The incidence of IF varies with geographical location with the lowest incidence reported in the United States being 0.012 cases per 1000 patients-day in 2007-2008 [2]. 

Disease-causing species are grouped into seven complexes with *Fusarium solani* and *Fusarium oxysporum* complexes causing the majority of IF [2]. Humans acquire infection by either direct inoculation into broken down skin, intravascular devices, or inhalation of conidia [3]. Disease severity is in part determined by the host’s immune system with localized infections such as keratitis, sinusitis, and cutaneous and nail infections occurring in immunocompetent individuals while disseminated IF occurs in immunocompromised patients, especially in the setting of neutropenia [3,4]. *Fusarium* virulence factors such as mycotoxins, enzyme production, and the ability to adhere to devices play important roles in the pathogenesis of the invasive disease [4]. Often, disseminated IF will manifest with persistent fever not responsive to broad-spectrum antimicrobial therapy in patients with malignancy-associated profound and prolonged neutropenia. Multiple organ systems are usually involved as reported in one case series with sinusitis occurring in 18%, and nodular or cavitary pneumonia in 39% [5] . Another study reported lung nodules in up to 82% on CT scans of patients with Fusariosis [2]. Endophthalmitis, central nervous system (CNS) infection, muscle, bone, and joint infections have also been reported [3]. Between sixty to seventy percent of patients with IF have cutaneous lesions [3,4]. The skin lesions are usually multiple erythematous subcutaneous nodules or painful erythematous macules and papules with necrotic centers [3]. Our patient had classic manifestations of IF with fever, skin, and pulmonary nodules and although a lung biopsy was not performed, the nodular lung lesions were presumed to be due to IF. 

The diagnosis of IF should always be considered in profoundly neutropenic patients with persistent fever. Blood and/or skin lesions biopsy cultures offer the best yield in reaching a diagnosis. Blood cultures are often positive in up to sixty percent of cases [3,4]. Blood cultures in fungal media will yield growth of *Fusarium *in a medium time of one to four days [2]. The colonies may appear red, grey, pink, or yellow on Sabouraud’s dextrose agar and microscopically *Fusarium *has a unique characteristic banana-shaped macroconidia [2]. Polymerase chain reaction (PCR) is quick and highly specific for the identification of *Fusarium*; however, sensitivity may vary depending on several factors including the type of specimen being tested, with tissue samples often yielding greatest sensitivity compared to body fluids such as BAL fluid [4,5]. Species identification by PCR is difficult and may require more sophisticated molecular methods such as multi-locus sequence typing (MLST) [ 4,5]. In histopathological tissue specimens, the presence of septate hyphae and characteristic canoe or banana-shaped conidia is suggestive of *Fusarium*  [3,5]. Its septate hyphae branch out in acute and right angles and can be seen on GMS or Periodic Acid-Schiff staining as well. Our patient’s skin biopsy GMS staining was suggestive of fungal hyphae and the culture grew *Fusarium sp* within four days. *Fusarium falciforme* was identified by a combination of phenotypic characteristics and DNA sequencing.

Differential diagnoses for IF include invasive aspergillosis (IA) which is more common than IF in immunosuppressed neutropenic patients and presents similarly with fever, pulmonary disease, and occasional skin lesions although blood cultures are rarely positive with IA [2]. Other disease-causing molds such as mucormycosis should be considered in the appropriate clinical context. With pulmonary disease, mycobacterial and dimorphic fungal infections should always be considered in the appropriate epidemiological and clinical setting. 

Treatment is based on expert opinion, experience, and case studies [1,4]. *Fusarium* *sp* are relatively intrinsically resistant to many antifungals especially echinocandins, and older azoles although various species may exhibit various susceptibility patterns [2,4]. Data to correlate minimum inhibitory concentrations (MICs) with clinical outcomes is lacking [4]. Amphotericin B and/or voriconazole are the most active agents in most cases, they are thus the preferred and/or alternative therapies respectively [2,4]. Given the variable susceptibility patterns, combination therapy should be considered despite the lack of supporting data for this approach. Combinations that have been reported in case studies include amphotericin B with voriconazole; amphotericin B with terbinafine; and voriconazole plus terbinafine [4]. There have been reports of synergy between voriconazole and terbinafine demonstrated in vitro. Intravenous therapy is preferred initially and then a switch to oral therapy can be considered depending on the patient’s clinical response and availability of oral antifungal options [2]. The duration of therapy is not defined however therapy should be continued until resolution of neutropenia, immune recovery, and a sustained clinical and radiological response is achieved [4]. Source control measures like surgical debridement of infected tissue, removal of infected devices, and catheters where applicable should accompany antifungal therapy [1]. Granulocyte and granulocyte-macrophage colony-stimulating factors should be used to aid neutrophil recovery [2,4]. 

Prognosis is poor with overall mortality rates in disseminated Fusariosis reported being between sixty to eighty percent in immunocompromised patients [2]. Prevention measures could be key in efforts to reduce this high mortality. All efforts should be made to prevent exposure in neutropenic patients. At-risk patients should always be placed in neutropenic isolation rooms with high-efficiency particulate air (HEPA) filters or positive pressure. Additionally, effort should be made for the patient to avoid contact with tap water and protect against skin breakdown. Providers should always perform a keen skin, hair, and nail examination looking for early skin lesions that could indicate IF. Prompt treatment of onychomycosis and surgical debridement of infected wounds should always be considered prior to start anticancer treatment [3,4]. In addition, delaying or reducing immunosuppression in at-risk patients can be considered [4]. 

## Conclusions

Fusariosis is an increasingly recognized cause of high morbidity and mortality among profoundly neutropenic patients with hematological malignancy and HSCT. IF should always be thought about in equal measure with IA. The infection should be urgently excluded in any patient with profound prolonged neutropenia and, if diagnosed, aggressive treatment should be immediately initiated. Early consultation with experts with experience in treating IF is of utmost importance as there are no guidelines for standard therapy. Adjunctive and preventive measures may have roles in reducing the high mortality in at-risk populations 
